# Real‐World Multinational Survey of Chronic Inflammatory Demyelinating Polyneuropathy: Disease Characteristics and Therapeutic Landscape

**DOI:** 10.1111/jns.70047

**Published:** 2025-08-18

**Authors:** Luis Querol, Simon Rinaldi, Andras Borsi, Giorgio Maria Boggia, Jonathan de Courcy, Yasmin Taylor, Jack Wright, Wisam Karmous, Wim Noel, Charlotte Gary, Gerd Meyer zu Hörste

**Affiliations:** ^1^ Hospital de la Santa Creu i Sant Pau Barcelona Spain; ^2^ Nuffield Department of Clinical Neurosciences University of Oxford Oxford UK; ^3^ Janssen‐Cilag Ltd, A Johnson & Johnson Company High Wycombe UK; ^4^ Janssen‐Cilag SpA, A Johnson & Johnson Company Milan Italy; ^5^ Adelphi Real World Bollington UK; ^6^ Janssen‐Cilag, A Johnson & Johnson Company Strasbourg France; ^7^ Janssen Pharmaceutica NV, A Johnson & Johnson Company Beerse Belgium; ^8^ University Hospital Münster Münster Germany

**Keywords:** chronic inflammatory demyelinating polyneuropathy, disease burden, healthcare resource utilization, patient reported outcomes, treatment patterns

## Abstract

**Background and Aims:**

Chronic inflammatory demyelinating polyneuropathy (CIDP) is an immune‐mediated syndrome characterized by progressive muscle weakness and sensory impairment. Clinical similarities with other neuropathies can cause misdiagnoses and delayed diagnoses. Additionally, a large proportion of patients appropriately treated according to current guidelines still show residual disability. This real‐world study aimed to characterize a global cohort of patients with CIDP.

**Methods:**

Data were drawn from the Adelphi CIDP Disease Specific Programme, a cross‐sectional survey with retrospective data collection, conducted in China, France, Germany, Italy, Japan, Spain, the United Kingdom, and the United States between September 2022 and April 2023. Neurologists and neuromuscular specialists reported on patient demographic and clinical characteristics at the time of the survey. Patients self‐reported treatment satisfaction, disease control, and health‐related outcome measures.

**Results:**

Overall, 164 physicians provided data for 1056 patients, with 428 (40.5%) providing self‐reported data. Patients were diagnosed with typical CIDP (69.2%) and variant CIDP (30.8%). Overall, initial misdiagnosis occurred in 37.2% of patients, with a median (interquartile range) diagnostic delay of 6.0 (3.0–12.0) months. Maintenance therapy was prescribed for 81.6% of patients, with corticosteroid use ranging from 25.7% in the United States to 80.0% in China. Some patients were dissatisfied by treatment outcomes (11.0%) and symptom control (12.2%). Overall, mean (SD) patient‐reported scores were 62.1 (20.4) for I‐RODS, 35.0 (11.1) for FACIT fatigue, and 0.662 (0.253) for EQ‐5D‐5L.

**Interpretation:**

Diagnostic delay and misdiagnoses were common occurrences across typical CIDP and variant CIDP. Despite the use of guideline treatments, there were unmet needs and a continued disease burden for patients.

## 1 Introduction

Chronic inflammatory demyelinating polyneuropathy (CIDP) is the most frequent form of chronic immune‐mediated polyneuropathy. Reported prevalences for CIDP show considerable regional variation, with an estimated range of 1–9 per 100 000 people [1]. CIDP is caused by primary immune‐mediated demyelination and secondary axonal damage in the peripheral nervous system, resulting in progressive muscle weakness and sensory impairment that led to significant disability [2]. CIDP can be delineated into typical CIDP, or a CIDP variant such as distal, multifocal, focal, motor, or sensory CIDP [3, 4].

Even with published diagnostic guidelines available [5], delayed diagnosis and misdiagnoses are common due to the rarity of CIDP, clinical similarities with other neuropathies, a lack of diagnostic biomarkers, and clinical variation. A retrospective Dutch study showed a median (range) 9 (1–334) month diagnostic delay in patients diagnosed outside of the tertiary referral center, and a 24 (2–190) month delay for those referred to the tertiary center with another diagnosis [6]. Cross‐sectional quantitative survey data from neurologists in the US found a majority did not make use of published guidelines in the diagnosis or treatment of CIDP [7]. Patients who experience misdiagnosis may be inadvertently subject to unnecessary costly treatment, which can result in burdensome adverse events (AEs) [6, 8, 9].

First‐line treatment options for CIDP are intravenous immunoglobulin (IVIg), corticosteroids, and plasma exchange treatment (PLEX). First‐line IVIg compared to prednisolone has shown little or no differential effect on disability or frequency of serious AEs [10]; PLEX also likely has a similar short‐term effect on disability when compared to IVIg. Patients' refractory or not responding to first‐line treatments may receive immunosuppressive drugs (e.g., azathioprine, ciclosporin, mycophenolate mofetil) or escalation therapies such as cyclophosphamide or rituximab [5]. However, due to the uncertain benefit of these second‐line therapies and the possibility of AEs, their use must be carefully discussed between the patient and physician regarding potential outcomes [5, 11]. Beyond this, novel treatment classes for CIDP, such as B‐cell inhibitors, complement inhibitors, and neonatal fragment crystallizable receptor (FcRn) blockers, are currently being investigated in clinical trials [12], with a FcRn blocker recently receiving US Food and Drug Administration (FDA) and European Medicines Agency (EMA) approval for the treatment of CIDP in 2024 and 2025, respectively [13, 14].

Due to its symptomatology, treatment options, and potential for misdiagnosis, CIDP is known to have a high physical and psychosocial burden [4]. Residual disability in patients with CIDP has been shown in several studies, with a recent meta‐analysis finding a majority of patients with long‐term CIDP still experiencing disability [15]. In a prospective Dutch cohort investigating clinical outcomes after 1 year of treatment, a majority of patients reported residual symptoms or deficits, irrespective of starting treatment [16]. Similar to this, a recent retrospective study on a UK cohort found residual disability in more than 60% of patients despite responding to treatment [17].

There is a lack of evidence assessing the treatment needs and burden of patients with CIDP from both the physician and patient perspective in a real‐world scenario. Additionally, there is limited data comparing patient and physician reported treatment satisfaction and disease control. This real‐world analysis aimed to characterize a global cohort of patients with CIDP and to identify unmet needs among them.

## 2 Methods and Materials

### 2.1 Study Design

This study was a secondary analysis of data drawn from the Adelphi Real World CIDP Disease Specific Programme (DSP), a cross‐sectional survey with elements of retrospective data collection, conducted in China, Europe (France, Germany, Italy, Spain, UK), Japan, and the USA between September 2022 and April 2023.

DSPs are surveys of physicians and their patients who present in a real‐world clinical setting and collect data on current disease management, disease‐burden impact, and associated treatment effects, using both clinical measures and the perspective of physicians and their patients. The DSP methodology has been previously described [18], validated [19], and demonstrated to be representative and consistent over time [20], countries, and disease areas, such as neurology [21].

### 2.2 Study Eligibility

Neurologists and neuromuscular specialists were recruited to participate in the DSP by local fieldwork agents, following completion of a short screening questionnaire. Physicians were eligible to participate in this survey if they were personally responsible for treatment decisions and management of patients with CIDP. It should be noted that neurologists can specialize in treating CIDP without becoming neuromuscular specialists, and that the neuromuscular specialist role is not universal across all countries. Physician participation was financially incentivized, with reimbursement upon survey completion according to fair market value.

Physicians were instructed to complete a patient record form for up to 10 eligible consecutively consulting patients who visited them for routine care, to mitigate against selection bias and to provide a patient sample representative of those presenting in a real‐world clinical setting. Patients were eligible for inclusion in the survey if they were 18 or older, had a physician‐confirmed diagnosis of CIDP, and visited the physician for consultation. Specified diagnostic criteria did not form part of this survey, and physician‐confirmed diagnoses did not undergo additional validation. However, the tests and assessments that were conducted to aid in patient CIDP diagnoses are given in the supplementary information. Record forms were completed at the time of their most recent consultation, collecting cross‐sectional data as well as retrospective information derived from historical clinical records.

### 2.3 Physician‐Reported Data

Upon recruitment, physicians completed an attitudinal survey to investigate their specialty and level of experience, practice setting, treatment practices, and perceptions about CIDP management.

The patient record form contained detailed questions on clinical and demographic characteristics, including symptomatic burden, CIDP variant, diagnostic delay (defined as time from symptom onset to physician confirmed diagnosis of CIDP), misdiagnosis, treatment patterns, and outcome satisfaction, and disease control, which was answered based on the physician's experience and clinical judgment. Comorbidities, such as anxiety and depression, could be selected from a predefined list from the Charlson comorbidities index; however, information on how they were defined or diagnosed was not part of the survey. Disease severity was measured using the Inflammatory Neuropathy Cause and Treatment (INCAT) Disability Score, where arm and leg problems were both scored 0–5 to give a total 0–10 score, spanning no disability to complete functional loss [22].

Patient record forms were completed with reference to patient clinical records to mitigate recall bias. This survey was designed to facilitate understanding of real‐world clinical practice, and thus physicians completed questions using their own clinical judgment and data they had to hand at the time of the consultation, representing the evidence available when making management decisions at that consultation. No additional diagnostic procedures, treatments, or other investigations were performed as part of this survey. Upon record form completion, physicians were compensated for participation according to fair market rates.

### 2.4 Patient‐Reported Data

Each patient for whom the physician completed a record form was invited to voluntarily complete a patient self‐reported questionnaire. Patient self‐reported questionnaires were completed by the patient independently from their physician and were returned in a sealed envelope, ensuring the patient's responses were kept confidential from their physician.

Patient‐reported questionnaires contained detailed questions including treatment satisfaction, disease control, as well as several patient‐reported outcome measures (PROMs). Patients were not compensated for participation.

### 2.5 Patient Reported Outcome Measures

Several measures were used to assess the outcomes of patients diagnosed with CIDP. Patient‐reported level of disability was measured using the Inflammatory Rasch‐built Overall Disability Scale (I‐RODS) [23]. In this study, scores were reported as centiles, with higher scores indicating lesser disability.

The 13‐item fatigue subscale of the Functional Assessment of Chronic Illness Therapy (FACIT) measurement system (Version 4.0) [24] provided a score for health‐related quality of life (HRQoL) in relation to fatigue. Higher scores indicate less fatigue and its subsequent impact upon daily activities, with a score range of 0 to 52. Scores of ≤ 21 were considered indicative of severe fatigue [25].

The EuroQol–5 dimension–5 level (EQ‐5D‐5L) health utility system was used to assess patients generic HRQoL [26, 27]. In line with guidance from the National Institute for Health and Care Excellence (NICE) [28], the study calculated utility values by mapping the 5 L descriptive system data onto the 3 L using the Hernandez‐Alava UK value set [29, 30]. These scores exist in the range of < 0 (worse than death), 0 (death) and 1 (perfect health) [29].

### 2.6 Ethical Considerations

Patients provided their informed consent to take part in the survey, and no identifiable protected health information was collected during the course of the survey. Data were anonymized and aggregated before being shared with the study team and/or for analysis and publication, thus collected in such a way that patients and physicians could not be identified directly.

Data collection was undertaken in line with European Pharmaceutical Marketing Research Association guidelines and, as such, did not require ethics committee approval [31]. The survey materials were submitted and reviewed by the PEARL International Review Board (IRB) (protocol #22‐ADRW‐153) and were approved for exempt research determination in accordance with the applicable federal regulations from approval.

In addition, each survey was performed in full accordance with relevant legislation at the time of data collection, including the US Health Insurance Portability and Accountability Act 1996 [32] and Health Information Technology for Economic and Clinical Health Act legislation [33].

### 2.7 Data Analysis

Descriptive analysis was performed on data from all study countries and/or the Europe region. Means and standard deviations, or medians and interquartile ranges, were calculated for continuous variables, and frequency counts and percentages for categorical variables.

All analyses were conducted in UNICOM Intelligence Reporter version 7.5.1 (UNICOM Systems 2021). Missing data were not imputed; therefore, the base of patients for analysis could vary from variable to variable and was reported separately for each analysis.

## 3 Results

### 3.1 Study Population

Overall, 164 neurologists completed patient record forms for 1056 of their consulting adult patients with CIDP. Of these patients, 428 (40.5%) completed the voluntary patient‐reported questionnaire.

Of the participating physicians, 82.1% were general neurologists and 17.9% had the specific title of neuromuscular specialist. Overall, physicians reported that they managed a median (IQR) of 20 (10–50) adult patients with CIDP each over the past 12 months prior to data collection. According to physicians' typical caseload, patients were consulted in either a public (hospital: 54.2%; office: 13.7%) or private setting (hospital: 14.0%; office: 18.0%). In addition, 65.2% reported spending at least some of their time on academic/teaching duties.

### 3.2 Patient Demographics and Clinical Characteristics in Accordance With Expectations

Data for patient demographics in Europe (*n* = 542, 51.3%), US (*n* = 291, 27.6%), China (*n* = 120, 11.4%), and Japan (*n* = 103, 9.8%) are shown in Table 1.

**TABLE 1:** Patient demographics & clinical characteristics split by region/country.

	All patients, (*n* = 1056)	Europe, (*n* = 542)	US, (*n* = 291)	China, (*n* = 120)	Japan, (*n* = 103)
Age (years), mean (SD)	54.0 (13.0)	54.0 (12.4)	54.5 (12.0)	47.4 (13.9)	59.8 (14.5)
Sex (male), *n* (%)	654 (61.9%)	337 (62.2%)	181 (62.2%)	66 (55.0%)	70 (68.0%)
BMI (kg/m^2^), mean (SD)	24.8 (3.5)	25.1 (3.1)	26.0 (3.7)	22.9 (2.9)	22.5 (3.3)
Number of concomitant conditions^a^, mean (SD)	0.9 (1.1)	0.8 (1.1)	1.1 (1.1)	1.0 (1.1)	0.6 (0.8)
Comorbidities, *n* (%)					
None shown	474 (44.9%)	271 (50.0%)	101 (34.7%)	49 (40.8%)	53 (51.5%)
Anxiety	140 (13.3%)	71 (13.1%)	61 (21.0%)	8 (6.7%)	0 (0.0%)
Depression	137 (13.0%)	72 (13.3%)	56 (19.2%)	4 (3.3%)	5 (4.9%)
INCAT, median (IQR)	3.0 (2.0–5.0)	3.0 (2.0–4.0)	2.0 (2.0–5.0)	4.0 (2.0–5.0)	3.0 (1.0–5.0)

Abbreviations: BMI, Body mass index; INCAT, Inflammatory Neuropathy Cause and Treatment; IQR, interquartile range; SD, Standard deviation.

^a^ The choices given to physicians for specific concomitant conditions also included: diabetes without chronic complications, chronic pulmonary disease, rheumatologic disease, peptic ulcer disease, mild liver disease, peripheral vascular disease, cerebrovascular disease, myocardial infarction, renal disease, monoclonal gammopathy (MGUS), congestive heart failure, diabetes with chronic complications, any malignancy, including leukemia and lymphoma, dementia, hemiplegia or paraplegia, metastatic solid tumor, long‐term effects of covid, moderate or severe liver disease, AIDS/HIV, and other.

Overall, at the time of the survey, the mean (SD) patient age was 54.0 (13.0) years, and 61.9% of patients were male, which ranged from 47.4 (13.9) years and 55.0% male in China to 59.8 (14.5) years and 68.0% male in Japan. Patients had a mean BMI of 24.8 (3.5), which ranged from 22.5 (3.3) in Japan to 26.0 (3.7) in the US (Table 1). At the time of the survey, patients had a median (IQR) disease duration of 3.3 (1.7–6.0) years.

The average number of concomitant conditions in patients was 0.9 (1.1), with anxiety reported at 13.3%, followed by depression at 13.0%. For the overall cohort, 55.1% had at least one concomitant condition, ranging from 65.3% in the US to 48.5% in Japan (Table 1).

**FIGURE 1:**
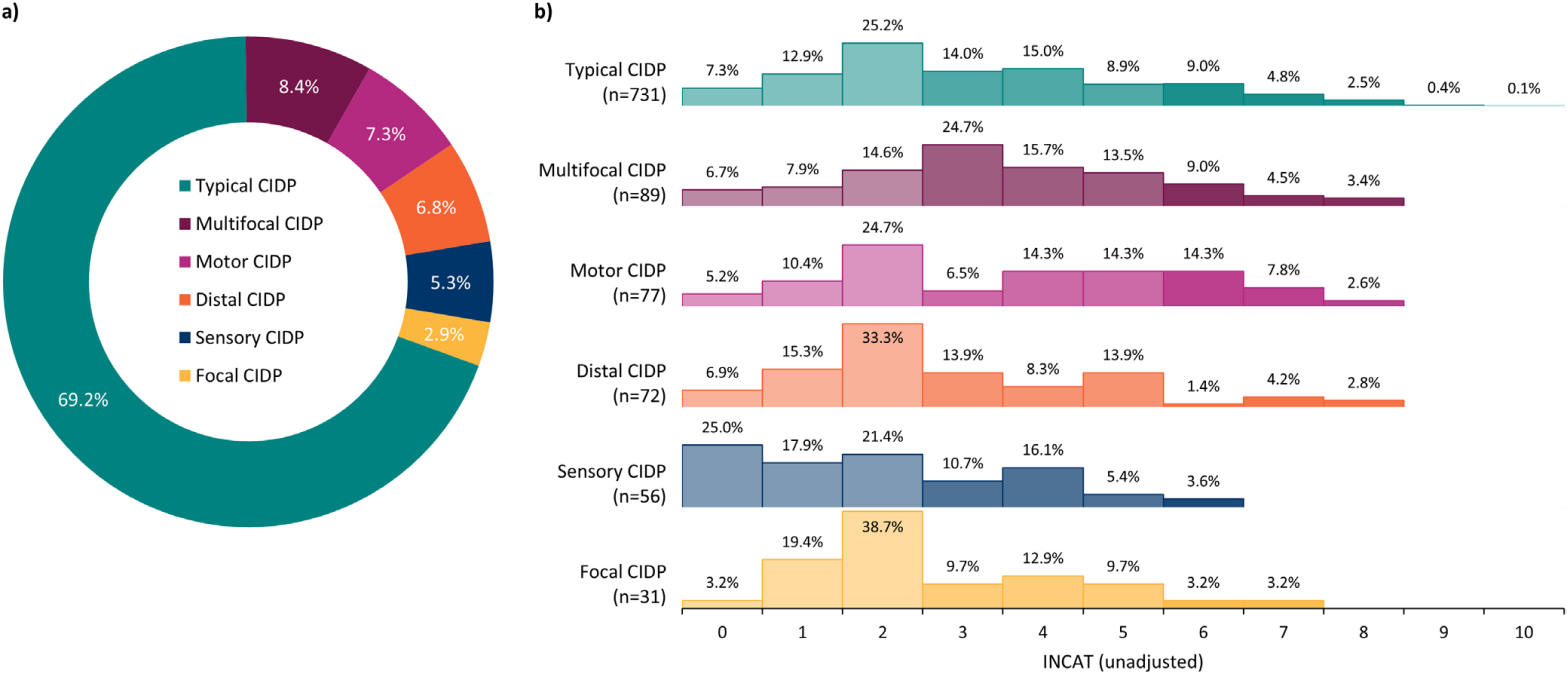
(a) frequency of CIDP variants within the overall patient sample; (b) distribution of INCAT scores across CIDP variants. Abbreviations: CIDP—Chronic inflammatory demyelinating polyneuropathy, INCAT—Inflammatory Neuropathy Cause and Treatment. ^1^The INCAT disability score, where 0 represents no disability and 10 represents complete functional loss.

A majority of patients were diagnosed with typical CIDP (69.2%). The most commonly reported variant overall was multifocal CIDP at 8.4% (Figure 1a). Between CIDP variants, there was variation in the distribution of INCAT scores. The proportions of those with a score of 3 or more ranged from 35.7% in sensory CIDP to 70.8% in multifocal CIDP. For a score of 6 or more, the range was 3.6% in sensory CIDP to 24.7% in motor CIDP (Figure 1b).

For symptoms experienced as an overall cohort, peripheral numbness was the most commonly reported at 70.5%, ranging from 67.4% in the US to 79.6% in Japan. The following two commonly reported symptoms had larger ranges between regions, with distal muscle weakness ranging from 42.3% in the US to 75.7% in Japan, and peripheral tingling ranging from 21.7% in China to 64.4% in Europe (Supplementary Table 1).

### 3.3 Diagnostic Journey Was Lengthy in Many Patients

**FIGURE 2:**
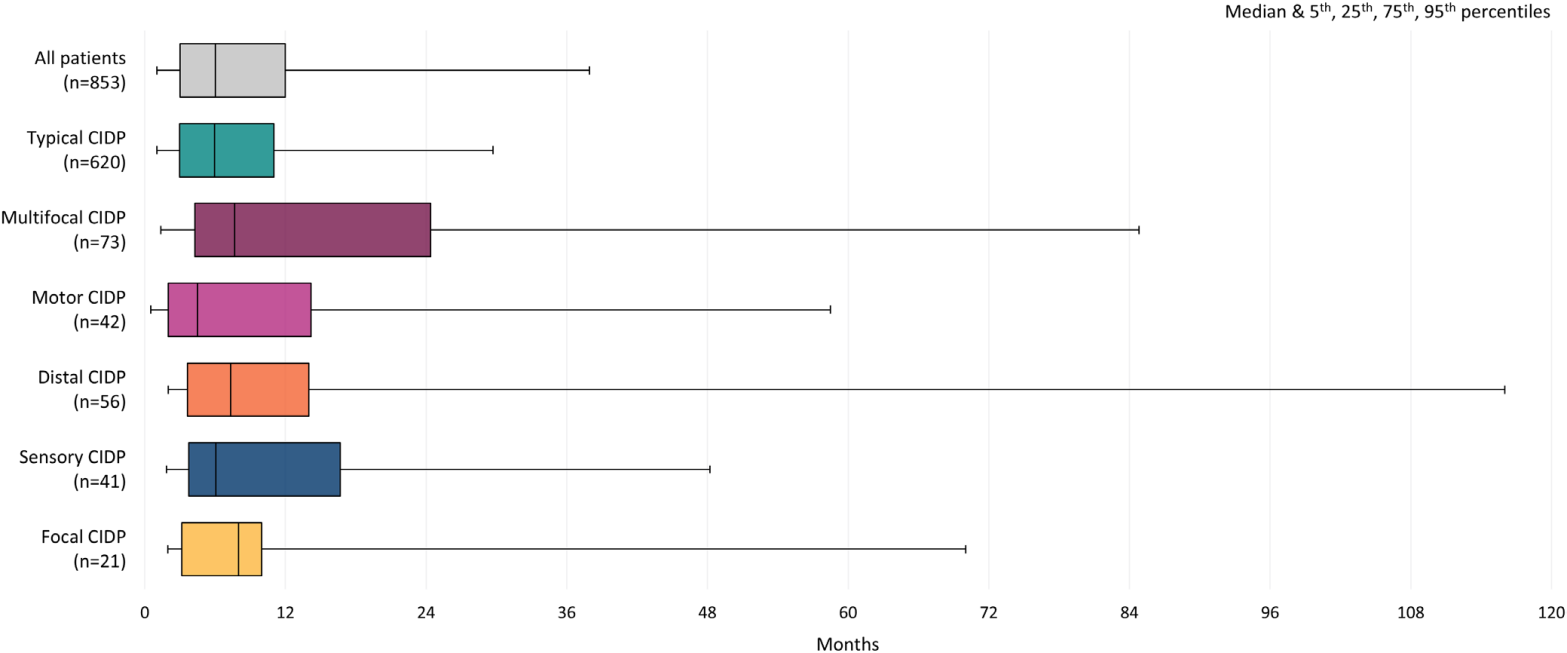
Diagnostic delay (months from symptom onset to diagnosis of CIDP) experienced by patients across CIDP variants. Abbreviations: CIDP—Chronic inflammatory demyelinating polyneuropathy. Diagnostic delay: Defined as time from symptom onset to physician‐confirmed diagnosis of CIDP.

Overall, 37.2% of patients were initially misdiagnosed or suspected to have another condition based on symptoms that were later attributed to CIDP. Between CIDP types, 35.6% with typical CIDP were initially misdiagnosed, and for those with variant CIDP, initial misdiagnosis ranged from 36.5% in motor CIDP to 44.0% in multifocal CIDP (Table 2). When Guillain‐Barré syndrome (GBS) was excluded, initial misdiagnosis for patients with typical CIDP occurred in 28.5%; and between variant CIDP, this ranged from 26.0% in typical CIDP to 36.8% in multifocal CIDP. Across all patients, the median (IQR) diagnostic delay was 6.0 (3.0–12.0) months, which ranged from 4.5 (2.0–14.2) months in motor CIDP to 8.0 (3.2–10.0) months in focal CIDP. The second longest reported diagnostic delay was multifocal CIDP at 7.7 (4.3–24.4) months (Table 2). The median diagnostic delay along with the 5th, 25th, 75th, and 95th percentiles, between CIDP variants are shown in Figure 2.

**TABLE 2:** Diagnostic journey split by CIDP variant.

	All patients	Typical CIDP	Distal CIDP	Multifocal CIDP	Focal CIDP	Motor CIDP	Sensory CIDP
Diagnostic delay (months)	*n* = 852	*n* = 620	*n* = 56	*n* = 73	*n* = 21	*n* = 41	*n* = 41
Median (IQR)	6.0 (3.0–12.0)	6.0 (3.0–11.0)	7.3 (3.6–14.0)	7.7 (4.3–24.4)	8.0 (3.2–10.0)	4.5 (2.0–14.2)	6.0 (3.7–16.7)
Initial misdiagnosis	*n* = 854	*n* = 599	*n* = 56	*n* = 75	*n* = 19	*n* = 63	*n* = 42
Yes	318 (37.2%)	213 (35.6%)	24 (42.9%)	33 (44.0%)	7 (36.8%)	23 (36.5%)	18 (42.9%)
Condition(s) misdiagnosed^a^, *n* (%)	*n* = 303	*n* = 204	*n* = 22	*n* = 32	*n* = 6	*n* = 21	*n* = 18
Guillain‐Barré syndrome (GBS)	87 (28.7%)	63 (30.9%)	6 (27.3%)	7 (21.9%)	0 (0.0%)	6 (28.6%)	5 (27.8%)
Diabetic polyneuropathy	43 (14.2%)	32 (15.7%)	3 (13.6%)	1 (3.1%)	1 (16.7%)	4 (19.0%)	2 (11.1%)
Multiple Sclerosis (MS)	36 (11.9%)	25 (12.3%)	2 (9.1%)	2 (6.3%)	0 (0.0%)	3 (14.3%)	4 (22.2%)
Fibromyalgia	31 (10.2%)	20 (9.8%)	1 (4.5%)	1 (3.1%)	0 (0.0%)	4 (19.0%)	5 (27.8%)
Toxic neuropathy	18 (5.9%)	13 (6.4%)	1 (4.5%)	2 (6.3%)	0 (0.0%)	1 (4.8%)	1 (5.6%)

*Note:* Diagnostic delay: defined as time from symptom onset to physician confirmed diagnosis of CIDP; Misdiagnosis: defined as initially diagnosed or suspected to have another condition responsible for symptoms that were later attributed to CIDP.

Abbreviations: CIDP—Chronic inflammatory demyelinating polyneuropathy, IQR—Interquartile range.

^a^ Other conditions misdiagnosed prior to confirmatory CIDP diagnosis included: amyotrophic lateral sclerosis (ALS), carpal tunnel syndrome, cerebral infarction, cervical spondylosis, diabetes complications, hereditary motor sensory neuropathy (HMSN), idiopathic polyneuropathy, lumbar disc herniation, lumbar spinal stenosis, lumbar spondylosis, Lyme disease, multifocal motor neuropathy (MMN), neuropathy, paraproteinemic neuropathy (PPN), peripheral nerve injury, peripheral neuropathy/neuritis, radiculopathy, rheumatoid arthritis, and other.

The most frequently reported conditions that formed a misdiagnosis, or were initially suspected to be causing patient symptoms, for all CIDP patients were GBS followed by diabetic polyneuropathy at 28.7% and 14.2%, respectively. Across all variants, aside from focal CIDP, GBS was ranked as the most reported misdiagnosed condition (Table 2). When GBS was excluded, the most frequently reported misdiagnosis or suspected condition was diabetic polyneuropathy (18.9%).

The majority of confirmatory CIDP diagnoses were made by a general neurologist for 78.2% of patients, ranging from 74.6% in Europe to 91.7% in Japan. Neuromuscular specialists were the diagnosing physicians for 21.2% of patients, ranging from 7.5% in China to 24.4% in Europe and the US. Less than 1% of patients were diagnosed by another healthcare professional (Supplementary Table 2).

The mean (SD) number of tests conducted to diagnose CIDP was 19.0 (9.8). Neurological examination was conducted in most patients (95.2%) to aid CIDP diagnosis. Overall, electromyograms and/or nerve conduction studies, nerve biopsies, and nerve ultrasound were used in the diagnosis of 90.8%, 17.7%, and 17.0% of patients, respectively. MRI and lumbar puncture/CSF testing were utilized in diagnosis for 53.9% and 63.6%, respectively (Supplementary Table 2).

### 3.4 Treatment History Featured High Complexity in CIDP Patients

**FIGURE 3:**
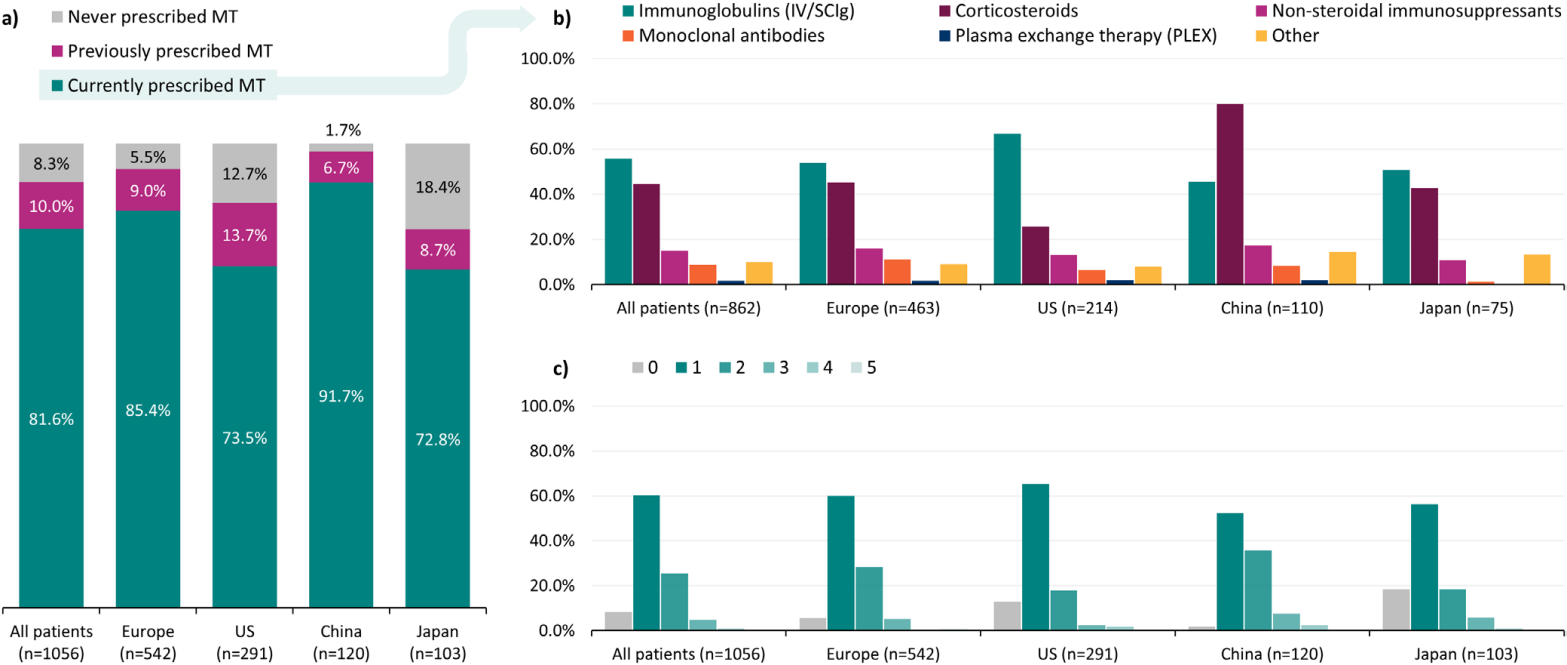
(a) patient maintenance treatment (MT) status at the time of the survey; (b) frequency of prescribed therapies among those receiving MT; (c) mean (SD) and frequency of lines of MT received; among the overall patient sample and across regions. Abbreviations: MT—Maintenance therapy, SD—Standard deviation. Due to 1 decimal place rounding, percentages do not total exactly 100% in 3a. Other maintenance treatments included: amifampridine, amitriptyline, carbamazepine, duloxetine, gabapentin, pregabalin, venlafaxine, other.

Patients' treatment status at the time of the survey is shown in Figure 3a. Maintenance therapy was prescribed for 81.6% of all patients at the time of the survey. In Europe, this was reported as 85.4%. In the US, China, and Japan, this was 73.5%, 91.7%, and 72.8%, respectively. Around a fifth (18.3%) of patients were not prescribed maintenance therapy at the time of the survey. The most common reason for patients to not be prescribed maintenance therapy was that their condition was stable without treatment, followed by the patient not wanting medication for their CIDP.

Among all patients, 55.7% were prescribed immunoglobulins (IV/SCIg) and 44.5% corticosteroids at the time of the survey. IV/SCIg prescription ranged from 45.5% in China to 66.8% in the US. Corticosteroid prescription ranged from 25.7% in the US to 80.0% in China. For the total cohort, there were only 14 reported uses of plasma exchange therapy. Non‐steroidal immunosuppressants, monoclonal antibodies, and other treatments, such as neuropathic pain therapies, were prescribed to 33.6% of patients (Figure 3b).

The majority of patients were prescribed a monotherapy; however, 31.2% of patients were prescribed more than one treatment at the time of data collection, ranging from 17.3% in Japan to 64.5% in China (Supplementary Table 2). For the overall patient cohort, it was reported that 60.2% of patients had received one line of therapy, 25.4% had received two lines, and 6.1% had received between 3 and 5 lines. There were also 88 (8.3%) patients that had no reported lines of therapy. The mean (SD) number of therapy lines was 1.3 (0.8), and in China it was 1.6 (0.8). The proportion of patients that received two lines of therapy ranged from 17.9% in the US to 35.8% in China (Figure 3c).

### 3.5 Physician and Patient Treatment and Symptom Control Satisfaction Was Limited in a Third of Patients

For the overall physician cohort, 82.7% reported that they were satisfied (“very satisfied” or “somewhat satisfied”) and 4.9% dissatisfied (“somewhat dissatisfied” or “very dissatisfied”) with treatment outcomes. Symptom control achieved by treatment was considered well controlled (“very well controlled” or “well controlled”) by 72.2% of physicians and having poor control (“poorly controlled” or “very poorly controlled”) by 6.2% of physicians (Supplementary Table 3).

**FIGURE 4:**
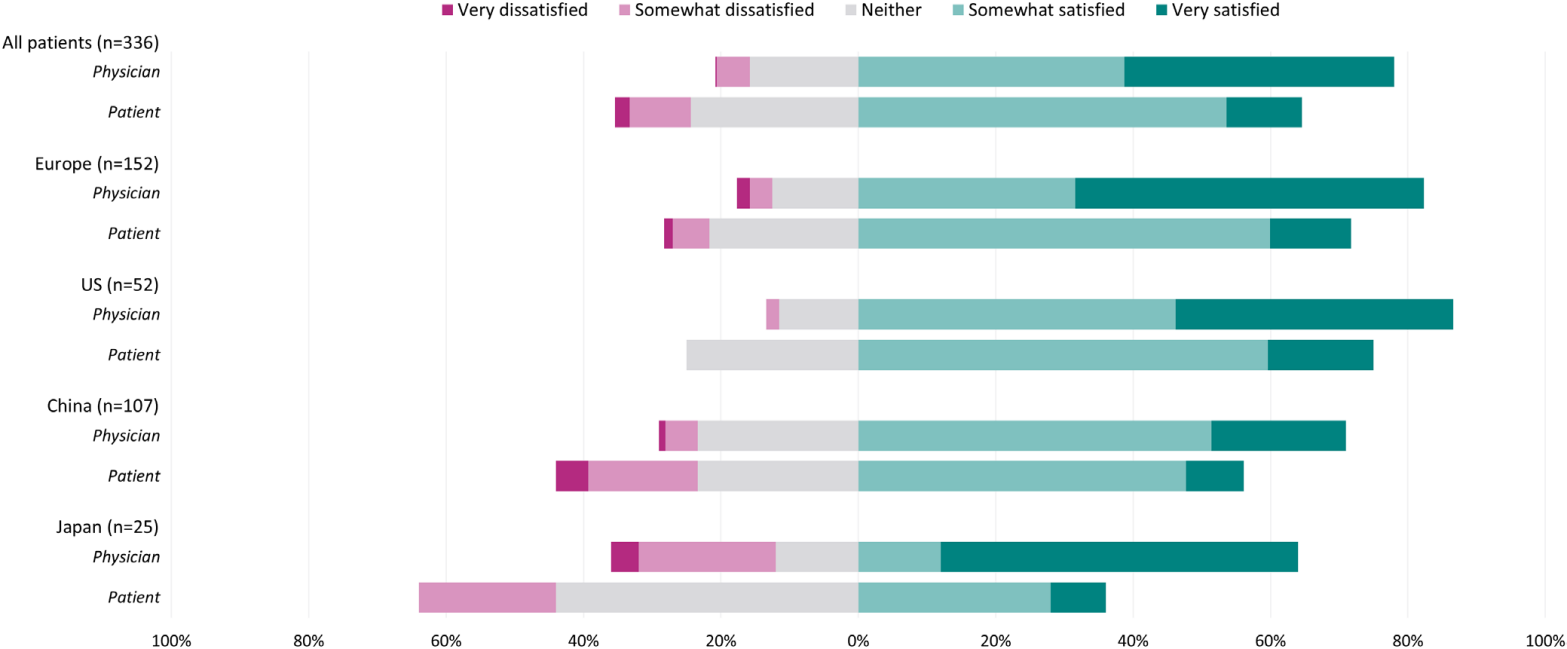
Overall satisfaction with current drug treatment, reported by matched physician‐patient dyads and split by region/country.

Among matched physician‐patient dyads, overall, 78.0% of physicians and 64.6% of patients reported that they were satisfied (“very satisfied” or “somewhat satisfied”) with treatment outcomes. Dissatisfaction (“somewhat dissatisfied” or “very dissatisfied”) with treatment outcomes were reported by 6.3% of physicians, and 11.0% of patients. These overall treatment satisfactions were reflected similarly in each region/country (Figure 4).

**FIGURE 5:**
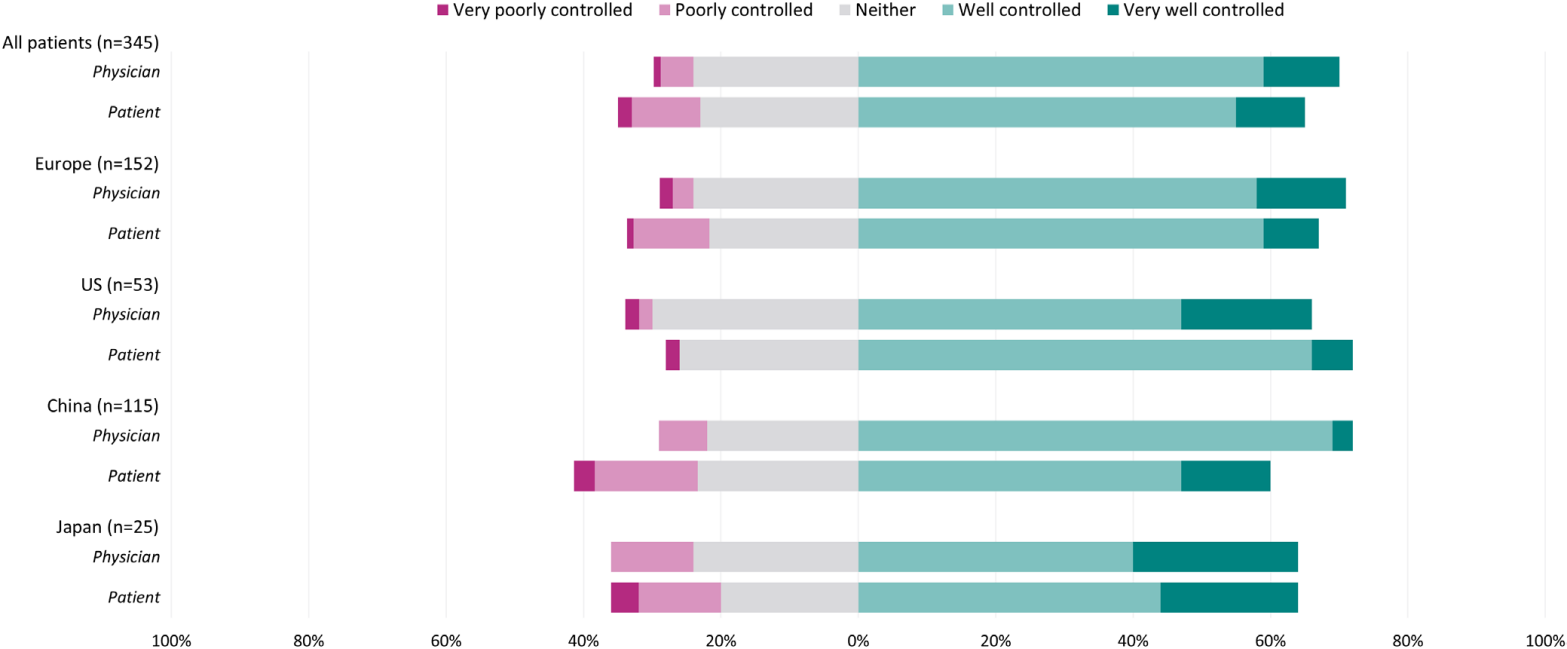
Perceived level of symptom control on current drug treatment, reported by matched physician‐patient dyads and split by region/country.

Overall, matched physician and patient reported levels of symptom control achieved by treatment were also recorded. Symptoms were considered well controlled (“very well controlled” or “well controlled”) by 69.9% of physicians and 64.9% of patients. For poor symptom control (“poorly controlled” or “very poorly controlled”), this was reported by 5.8% of physicians and 12.2% of patients. (Figure 5).

### 3.6 Health Care Resource Utilization Was Moderately High

**FIGURE 6:**
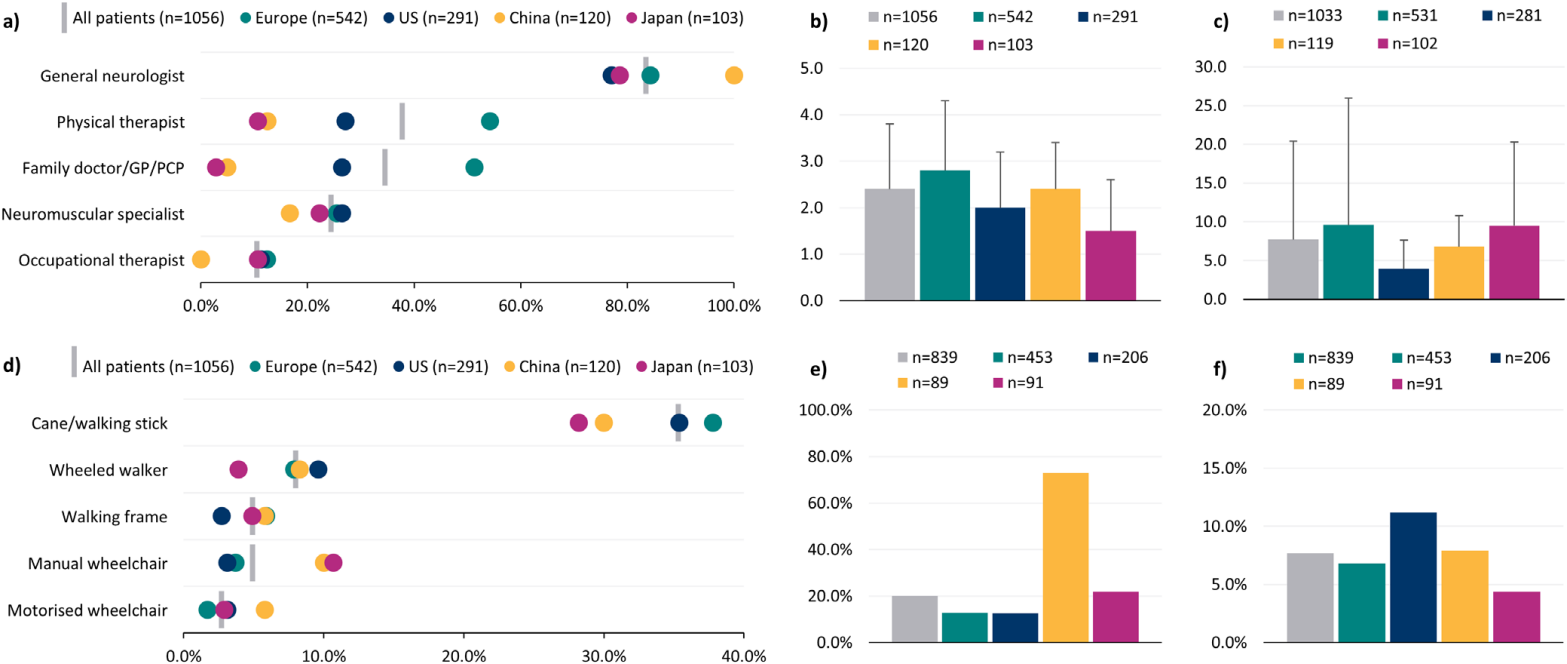
Health care resource utilization, a) frequency of HCP involvement in patient management (five most frequently involved HCPs shown); b) mean (SD) total HCP types involved in patient management; c) mean (SD) total HCP consultations in the 12 months prior to survey; d) mobility aid utilization (five most frequently utilized devices shown); e) percentage of patients with one or more hospitalization in relation to CIDP in the 12 months prior to survey; f) percentage of patients with one or more ER admissions in the 12 months prior to survey. Abbreviations: ER—Emergency room, GP—General practitioner, HCP—Health care professional, PCP—Primary care physician, SD—Standard deviation. HCPs that were involved in less than 10% of all patient management included: nurse, internal medicine/internist, rehabilitation therapist, psychologist, psychiatrist, ophthalmologist, physician associate/assistant, speech and language therapist, neuropsychiatrist, and other. Mobility aids that were utilized by fewer than 2% of all patients included: Modified car (e.g., wheelchair accessible vehicle), motorized scooter, and other.

The top three most reported health care professionals (HCPs) involved in patient management were general neurologists (83.5%), followed by physical therapists (37.8%) and family doctor/general practitioner/primary care physician (34.5%). In China, 100% of patients were reported to have received care management from general neurologists, 16.7% from neuromuscular specialists, 12.5% from physical therapists, and 5.0% from family doctor/general practitioner/primary care physician (Figure 6a and Supplementary Table 4). A mean (SD) of 2.4 (1.4) HCPs were involved in patient management; in the 12 months prior to the survey, patients had a mean of 7.7 (12.7) HCP consultations (Figure 6b,c).

Overall, 47.3% of patients with CIDP (ranging from 41.7% in Japan and 50.0% in China) were recorded to use mobility aids at the time of data collection. Across all regions, a cane/walking stick was used by 35.3% of patients, followed by the wheeled walker (8.0%), walking frame, and/or manual wheelchair (both 4.9%) (Figure 6d **and** Supplementary Table 4).

Most patients (79.9%) did not have any hospitalizations in the 12 months prior to the time of survey. Over a tenth of patients (12.8%) had one hospitalization due to their CIDP, exclusive of treatment, and 7.5% had two or more hospitalizations. This was not shared by patients in China, where 27.0% were reported to have had no hospitalizations, and a proportion of single, double, and triple hospitalizations at 34.8%, 19.1%, and 13.5%, respectively (Figure 6e and Supplementary Table 4). Overall, 92.3% of patients did not have any ER admissions in the 12 months prior to the time of survey. The proportions for the number of ER admissions for patients in China were within the range of all patients (Figure 6f).

### 3.7 Patient Reported Outcome Measures Reflect High Disease Burden

The median (IQR) INCAT among the sample of patients with patient‐reported data was 2.0 (2.0–4.0), comparable to that observed among the overall sample.

**FIGURE 7:**
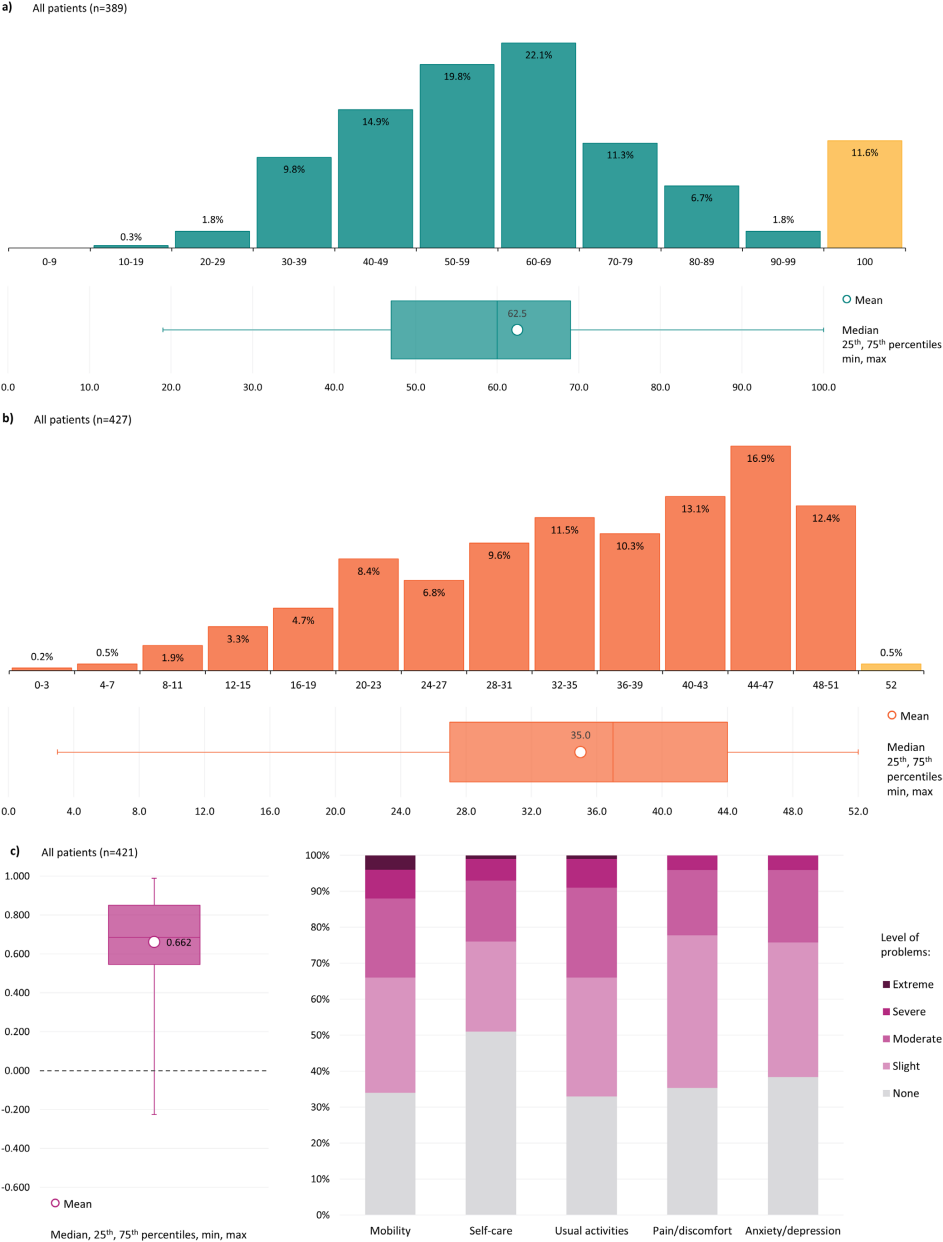
(a) Distribution of I‐RODS centile scores; (b) Distribution of FACIT‐Fatigue scores; (c) Distribution of EQ‐5D health utility values, and frequency of response levels to individual dimensions. FACIT—Functional Assessment of Chronic Illness Therapy. I‐RODS—Inflammatory Rasch‐built Overall Disability Scale. I‐RODS data has been presented as centiles, with higher centile scores indicating lower disability. For FACIT‐Fatigue, higher scores indicate lesser fatigue. The EQ‐5D‐5L health utility values can exist in the range of < 0 (worse than death), 0 (death) and 1 (perfect health).

This cohort reported a wide distribution of I‐RODS centile scores, with the mean (SD) score as 62.1 (20.4). The proportion of patients within each I‐RODS centile ranged from 22.1% between 60–69 and 0.3% between 10 and 19. There were no patients between the centile 0–9. In the 100th percentile, there were 11.6%, indicative of no disability (Figure 7a).

For all patients, the mean (SD) FACIT fatigue score was 35.0 (11.1). The majority of patients (52.7%) had scores between 36 and 51. Around one in ten (10.6%) scored between 0 and 19, indicative of severe fatigue (Figure 7b).

Overall, the mean (SD) EQ‐5D‐5L health utility score was 0.662 (0.253). The minimum–maximum range of patient scores was −0.225 to 0.989. Between individual EQ‐5D‐5L domains, there were similar response frequencies. Only self‐care had no issues (none) as a majority of responses (50.6%). In mobility and usual activities, 33.4% and 33.5%, respectively, had moderate to extreme issues (Figure 7c).

## 4 Discussion

We report on an international cohort of patients diagnosed with typical CIDP or variant CIDP. Overall, our data show that initial misdiagnoses for patients with CIDP remain a frequent occurrence. Additionally, even with roughly 80% of patients in our sample on maintenance therapy (mostly immunoglobulins and/or corticosteroids) at the time of survey, patient self‐reported data indicate the presence of residual disability.

Our cohort was the majority male, and most had at least one comorbidity, which aligns with previous studies [5, 34]. In relation to variants of CIDP, we found 69.2% had typical CIDP and 30.8% had variant CIDP, with proportions consistent with a recent meta‐analysis, which reported the rate of typical CIDP as 62% [15].

Initial misdiagnosis was common in both typical and variant CIDP, with our data showing frequent misdiagnosis in CIDP variants, consistent with current reporting [5, 9]. For our cohort overall, the total rate of misdiagnosis was more than a third, with GBS as the single most common misdiagnosis. In two single center studies, absolute rates of CIDP misdiagnosis ranged from 68% to 32%, and showed differing rates of GBS misdiagnosis, from 23% to 6% [6, 35].

Misdiagnosis also contributes to diagnostic delay. In our cohort, the overall median delay in diagnosis was half a year, and there was apparent variation between CIDP variants, suggesting that the longest delays occur in focal and multifocal CIDP. Two previous single‐center studies seem to suggest longer delays occur when patients with CIDP are referred with non‐CIDP conditions from outside of specialist centers, specifically for patients with an initial non‐GBS referral [6, 35]. It is likely that between countries and regions, the rates of misdiagnosis and the associated diagnostic delays will vary. While misdiagnoses can only partially explain delayed diagnoses, it is of importance to understand why, in some cases, these rates can be so high and how variant CIDP can affect this.

Similar proportions of patients were prescribed immunoglobulin therapy within each region; however, there were apparent variations in the proportion of patients prescribed corticosteroids. In China, 80% of patients were reported to be prescribed corticosteroids, over 40% were prescribed immune suppressants or other therapies, and a majority of patients received one or two lines of any therapy combination. The long‐term use of corticosteroids has been associated with AEs in patients with CIDP [36], and there is sparse data for the effectiveness of immune suppressants or other therapies [5].

Additionally, our data showed that a majority of patients in China had one or more hospitalizations in the last 12 months. A real‐world study of patients with CIDP in China noted that intravenous immunoglobulin therapy has limited availability and high cost, further suggesting that some patients may start IVIg treatment but are not able to complete a recommended course, leading to symptom relapse [37]. A randomized, double‐blind trial in the Netherlands showed that safe withdrawal from IVIg using a weaning period is possible and is suggested for clinically stable patients with CIDP [38]. Further research into the impacts of sudden treatment withdrawal could be of value in countries with less predictable access to specific treatments.

From our matched physician‐patient self‐reported data, disconnect was observed for overall treatment satisfaction, in which it appears that physicians more frequently indicated higher levels of satisfaction than patients, and synonymously patients more commonly reported levels of treatment dissatisfaction. This was similar in terms of symptom control, although less pronounced. Patients indicating treatment dissatisfaction may be influenced by burdens associated with the administration of treatment, having to travel to a health center, and receiving injections. These factors may have less of an impact on physician‐reported satisfaction. It is important that patients feel they can ask their physician for treatment alternatives if they are dissatisfied. A recent meta‐analysis found that independence and flexibility provided by subcutaneous immunoglobulins are preferential and that patients experienced comparable health outcomes to IVIg [39]. Treatment individualization based on patient preferences, disease behavior, and minimally effective immunoglobulin doses could be key steps to improve patient treatment satisfaction.

Patient reported outcome measures (PROMs) in our study indicate high levels of residual disability in the cohort overall. Patient I‐RODS scores are indicative of residual disability based on self‐reported impairment to activities of daily living. High levels of fatigue can be inferred by comparison to German FACIT norms, which were reported to be a mean (SD) of 43.5 (8.3) [40]. Levels of moderate or worse outcomes in EQ‐5D‐5L were reported for patients in each of the five domains. Based on mean EQ‐5D‐3L norms in the UK (0.856) and mean EQ‐5D‐5L norms in Germany (0.88), patients experience lower HRQoL in comparison to the general population [40, 41]. While there are potential issues with the I‐RODS regarding its questions' generalizability between countries and patient understanding, validated and disease‐specific PROMs represent an important tool in the dialog between neurologists and patients, improving patient care and satisfaction [42]. Our FACIT and EQ‐5D‐5L data suggest there exists significant unmet medical need in patients with CIDP. Additionally, the presence of concomitant conditions may further add to patient burden due to associated treatment complications, an important consideration in a population that seemingly tends to have one or more comorbidities [34].

Patient satisfaction and PROMs point to the need for novel CIDP targeted treatments. FcRn blockers have begun to show promising results for patients with CIDP, with a recent FDA and EMA approval [13, 14] and another anti‐FcRn monoclonal antibody currently being evaluated in a phase 3 clinical trial [43]. Complement pathway inhibitors also offer another possibility for the targeted treatment of patients with CIDP, with a novel inhibitor being shown to have a favorable benefit/risk profile from phase 2 clinical trial data [44] and now moving forward in two phase 3 clinical trials [45, 46]. As these therapies enter clinical practice, it will be important to assess their real‐world effectiveness.

## 5 Strengths and Limitations

This study had a number of strengths and limitations. While minimal inclusion criteria governed the selection of the participating physicians, who were recruited to provide a geographically diverse and pragmatic sample, participation was influenced by willingness to complete the survey. The large and multinational cohort of physicians and patients included in this study aids the generalizability of the results. The cross‐sectional design of this study prevents any conclusions about causal relationships; however, the identification of significant associations was possible.

While physicians are asked to provide data for a consecutive series of patients to avoid selection bias, patients who visit their physician more frequently may be more severely affected than those with mild disease who do not consult their physician as frequently.

CIDP diagnosis was not tied to specified diagnostic criteria or additional validation, but used the presence of a physician‐confirmed diagnosis of CIDP in patient medical records, reflective of real‐world practice. This helped to provide a more holistic picture of the CIDP landscape in a multinational cohort where there may be local differences. The survey did ask physicians to report which tests and assessments were used in patient CIDP diagnosis, provided in (Supplementary Table 2).

While recall bias is a common limitation of surveys, the data for these analyses were collected at the time of each patient's appointment, which is expected to reduce this likelihood. In addition, physicians had access to patient medical records for data extraction.

Despite such limitations, real‐world studies play an important part in highlighting areas of concern that are not addressed in clinical trials, with the data in this study representative of current clinical practice at the time the survey was conducted.

## 6 Conclusions

There is evident disease burden for patients with CIDP across Europe, US, China, and Japan, despite the majority of patients being prescribed maintenance therapy, and the existence of second‐line treatment options. CIDP diagnostic delay and misdiagnoses are still common occurrences across CIDP variants. These findings may be of use to physicians in clinical practice who have CIDP patients that are not responding well to their current treatment.

### 6.1 Future Study Recommendations

Further studies into patient outcomes after an inadequate response to first‐line CIDP treatments are required. Additionally, as novel targeted treatments become more commonplace, it will be important to analyze patient outcomes in comparison to current practice.

## Disclosure

Luis Querol is an employee of Hospital de la Santa Creu i Sant Pau. Luis Querol received research grants from Instituto de Salud Carlos III—Ministry of Economy and Innovation (Spain), CIBERER, Fundació La Marató, GBS‐CIDP Foundation International, and ArgenX. Luis Querol received speaker or expert testimony honoraria from CSL Behring, Novartis, Sanofi‐Genzyme, Merck, Annexon, Alnylam, ArgenX, Dianthus, LFB, Avilar Therapeutics, Lycia Therapeutics, Nuvig Therapeutics, Takeda, and Roche. Luis Querol serves at Clinical Trial Steering Committees for Sanofi Genzyme, ArgenX, and Takeda and is Principal Investigator for UCB's CIDP01 trial and Sanofi's Mobilize and Vitalize trials.

Simon Rinaldi is an employee of Nuffield Department of Clinical Neurosciences, University of Oxford. He is currently supported by grants from the Medical Research Council (UK), GBS|CIDP Foundation International, and British Medical Association. He has previously been supported by grants from the Medical Research Council (UK), Wellcome Trust, National Institute of Health Research (NIHR), the Pathological Society of Great Britain and Ireland, and the University of Oxford's John Fell Fund. He has received honoraria for lectures given at the request of Excemed, Fresenius, CSL Behring, UCB, argenx, the Beijing Association of Holistic and Integrated Medicine, and the Irish Institute of Clinical Neuroscience. He has been a paid consultant for argenx, Annexon, Dianthus, Takeda, and Hansa Biopharma. He is an unpaid member of the medical advisory board of the Guillain‐Barré syndrome and Related Inflammatory Neuropathies (GAIN) charity and of the Inflammatory Neuropathy Consortium (INC) board. He has previously received reduced registration fees, travel grants, and scientific prizes from the Peripheral Nerve Society. He runs a not‐for‐profit diagnostic testing service for nodal/paranodal antibodies.

Gerd Meyer zu Hörste is an employee of University Hospital Münster. In the last 5 years, GMzH received speaker honoraria from Alexion, LFB Pharma, Amgen, Argenx; compensation for serving on advisory boards for LFB Pharma, Immunovant, Roche; and received project‐related research funding from Merck (Germany), Biogen, Roche. He received no compensation for his activity in this study.

Andras Borsi, Charlotte Gary, Wim Noel, Wisam Karmous, and Giorgio Maria Boggia are employees of Johnson & Johnson Innovative Medicine and may hold stock.

Jonathan DeCourcy, Yasmin Taylor, and Jack Wright are employees of Adelphi Real World.

## Supporting information


**Supplementary Table 1:** Symptomatology split by region/country.

**Supplementary Table 2:** Diagnosis and treatment split by region/country.

**Supplementary Table 3:** Physician reported overall satisfaction with, and perceived level of symptom control on, current drug treatment, and split by region/country.

**Supplementary Table 4:** Healthcare resource utilization split by region/country.

## Data Availability

The data that support the findings of this study are available from the corresponding author upon reasonable request.
