# Supplementary material for: Real‐World Multinational Survey of Chronic Inflammatory Demyelinating Polyneuropathy: Disease Characteristics and Therapeutic Landscape
Source: J Peripher Nerv Syst. 2025 Aug 18;30(3):e70047. doi: 10.1111/jns.70047 (PMC12361836; doi:10.1111/jns.70047)
Supplement: Supplementary file 1 — Supplementary Table 1: Symptomatology split by region/country. [file JNS-30-0-s003.docx]

## **Supplementary Table 1**: Symptomatology split by region/country

|  | **All patients**  **(n = 1056)** | **Europe**  **(n = 542)** | **US**  **(n = 291)** | **China**  **(n = 120)** | **Japan**  **(n = 103)** |
| --- | --- | --- | --- | --- | --- |
| **Clinical features at time of survey (top 10), n (%)** |  |  |  |  |  |
| Peripheral numbness | 744 (70.5%) | 375 (69.2%) | 196 (67.4%) | 91 (75.8%) | 82 (79.6%) |
| Distal muscle weakness | 590 (55.9%) | 335 (61.8%) | 123 (42.3%) | 54 (45.0%) | 78 (75.7%) |
| Peripheral tingling | 552 (52.3%) | 349 (64.4%) | 141 (48.5%) | 26 (21.7%) | 36 (35.0%) |
| Areflexia | 467 (44.2%) | 277 (51.1%) | 100 (34.4%) | 27 (22.5%) | 63 (61.2%) |
| Proximal muscle weakness | 414 (39.2%) | 229 (42.3%) | 76 (26.1%) | 54 (45.0%) | 55 (53.4%) |
| Neuropathic pain | 326 (30.9%) | 193 (35.6%) | 87 (29.9%) | 28 (23.3%) | 18 (17.5%) |
| Physical fatigue/low energy | 291 (27.6%) | 178 (32.8%) | 63 (21.6%) | 37 (30.8%) | 13 (12.6%) |
| Difficulty walking/maintaining gait | 289 (27.4%) | 173 (31.9%) | 60 (20.6%) | 30 (25.0%) | 26 (25.2%) |
| Loss of balance/falling | 246 (23.3%) | 146 (26.9%) | 69 (23.7%) | 26 (21.7%) | 5 (4.9%) |
| Peripheral burning | 227 (21.5%) | 119 (22.0%) | 80 (27.5%) | 13 (10.8%) | 15 (14.6%) |
