# Supplementary material for: Real‐World Multinational Survey of Chronic Inflammatory Demyelinating Polyneuropathy: Disease Characteristics and Therapeutic Landscape
Source: J Peripher Nerv Syst. 2025 Aug 18;30(3):e70047. doi: 10.1111/jns.70047 (PMC12361836; doi:10.1111/jns.70047)
Supplement: Supplementary file 2 — Supplementary Table 2: Diagnosis and treatment split by region/country. [file JNS-30-0-s001.docx]

## **Supplementary Table 2**: Diagnosis and treatment split by region/country

|  | **All patients** | **Europe** | **US** | **China** | **Japan** |
| --- | --- | --- | --- | --- | --- |
| **Diagnosing physician, n (%)** | n = 1054 | n = 540 | n = 291 | n = 120 | n = 103 |
| General neurologist | 824 (78.2%) | 403 (74.6%) | 219 (75.3%) | 110 (91.7%) | 92 (89.3%) |
| Neuromuscular specialist | 223 (21.2%) | 132 (24.4%) | 71 (24.4%) | 9 (7.5%) | 11 (10.7%) |
| Internal medicine/Internist | 4 (0.4%) | 2 (0.4%) | 1 (0.3%) | 1 (0.8%) | 0 (0.0%) |
| Other ^1^ | 3 (0.3%) | 3 (0.6%) | 0 (0.0%) | 0 (0.0%) | 0 (0.0%) |
| **Total number of tests to diagnose CIDP, n** | n = 1056 | n = 542 | n = 291 | n = 120 | n = 103 |
| Mean (SD) | 19.0 (9.8) | 22.3 (9.7) | 12.4 (8.2) | 17.5 (7.3) | 21.9 (7.5) |
| **Tests/assessments conducted to aid CIDP diagnosis, n (%)** | n = 1056 | n = 542 | n = 291 | n = 120 | n = 103 |
| General Tests |  |  |  |  |  |
| Neurological examination | 1005 (95.2%) | 537 (99.1%) | 276 (94.8%) | 89 (74.2%) | 103 (100.0%) |
| Review of medical history | 897 (84.9%) | 484 (89.3%) | 233 (80.1%) | 78 (65.0%) | 102 (99.0%) |
| Muscle and nerve tests ^2^ |  |  |  |  |  |
| Electromyogram (EMG) and/or Nerve conduction study | 959 (90.8%) | 530 (97.8%) | 226 (77.7%) | 104 (86.7%) | 99 (96.1%) |
| Somatosensory evoked potentials | 278 (26.3%) | 202 (37.3%) | 28 (9.6%) | 16 (13.3%) | 32 (31.1%) |
| Nerve biopsy | 187 (17.7%) | 119 (22.0%) | 33 (11.3%) | 21 (17.5%) | 14 (13.6%) |
| Nerve ultrasound | 180 (17.0%) | 98 (18.1%) | 16 (5.5%) | 44 (36.7%) | 22 (21.4%) |
| Blood tests ^3^ |  |  |  |  |  |
| Complete blood count | 793 (75.1%) | 446 (82.3%) | 168 (57.7%) | 95 (79.2%) | 84 (81.6%) |
| Liver function | 694 (65.7%) | 370 (68.3%) | 147 (50.5%) | 95 (79.2%) | 82 (79.6%) |
| Glycosylated haemoglobin (HbA1c) | 692 (65.5%) | 371 (68.5%) | 135 (46.4%) | 96 (80.0%) | 90 (87.4%) |
| Antinuclear antibody antibodies (ANA) | 687 (65.1%) | 397 (73.2%) | 127 (43.6%) | 82 (68.3%) | 81 (78.6%) |
| C-reactive protein | 682 (64.6%) | 399 (73.6%) | 102 (35.1%) | 91 (75.8%) | 90 (87.4%) |
| Anti-ganglioside antibodies (anti-GM1) | 675 (63.9%) | 423 (78.0%) | 90 (30.9%) | 85 (70.8%) | 77 (74.8%) |
| Creatine kinase | 639 (60.5%) | 369 (68.1%) | 107 (36.8%) | 75 (62.5%) | 88 (85.4%) |
| Fasting blood glucose | 638 (60.4%) | 368 (67.9%) | 97 (33.3%) | 88 (73.3%) | 85 (82.5%) |
| Scans and imaging ^4^ |  |  |  |  |  |
| MRI scan | 569 (53.9%) | 325 (60.0%) | 104 (35.7%) | 53 (44.2%) | 87 (84.5%) |
| CT scan | 247 (23.4%) | 148 (27.3%) | 20 (6.9%) | 55 (45.8%) | 24 (23.3%) |
| X-ray | 142 (13.4%) | 65 (12.0%) | 4 (1.4%) | 35 (29.2%) | 38 (36.9%) |
| Other ^5^ |  |  |  |  |  |
| Lumbar puncture/CSF testing | 672 (63.6%) | 381 (70.3%) | 123 (42.3%) | 77 (64.2%) | 91 (88.3%) |
| **Number of prescribed treatments at time of survey, n (%)** | n = 862 | n = 463 | n = 214 | n = 110 | n = 75 |
| Monotherapy | 593 (68.8%) | 317 (68.5%) | 175 (81.8%) | 39 (35.5%) | 62 (82.7%) |
| More than one treatment | 269 (31.2%) | 146 (31.5%) | 39 (18.2%) | 71 (64.5%) | 13 (17.3%) |
| Mean (SD) | 1.4 (0.7) | 1.4 (0.7) | 1.3 (0.6) | 1.8 (0.7) | 1.3 (0.8) |
