# Supplementary material for: Real‐World Multinational Survey of Chronic Inflammatory Demyelinating Polyneuropathy: Disease Characteristics and Therapeutic Landscape
Source: J Peripher Nerv Syst. 2025 Aug 18;30(3):e70047. doi: 10.1111/jns.70047 (PMC12361836; doi:10.1111/jns.70047)
Supplement: Supplementary file 3 — Supplementary Table 3: Physician reported overall satisfaction with, and perceived level of symptom control on, current drug treatment, and split by region/country. [file JNS-30-0-s004.docx]

## **Supplementary Table 3:** Physician reported overall satisfaction with, and perceived level of symptom control on, current drug treatment, and split by region/country

|  | **All patients** | **Europe** | **US** | **China** | **Japan** |
| --- | --- | --- | --- | --- | --- |
| **Overall satisfaction with current drug treatment, n (%)** | n = 862 | n = 463 | n = 214 | n = 110 | n = 75 |
| Very satisfied | 327 (37.9%) | 195 (42.1%) | 86 (40.2%) | 22 (20.0%) | 24 (32.0%) |
| Somewhat satisfied | 388 (45.0%) | 195 (42.1%) | 107 (50.0%) | 56 (50.9%) | 30 (40.0%) |
| Neither satisfied nor dissatisfied | 105 (12.2%) | 52 (11.2%) | 20 (9.3%) | 25 (22.7%) | 8 (10.7%) |
| Somewhat dissatisfied | 32 (3.7%) | 15 (3.2%) | 1 (0.5%) | 6 (5.5%) | 10 (13.3%) |
| Very dissatisfied | 10 (1.2%) | 6 (1.3%) | 0 (0.0%) | 1 (0.9%) | 3 (4.0%) |
| **Perceived level of symptom control on current drug treatment, n (%)** | n = 968 | n = 512 | n = 254 | n = 118 | n = 84 |
| Very well controlled | 143 (14.8%) | 70 (13.7%) | 50 (19.7%) | 3 (2.5%) | 20 (23.8%) |
| Well controlled | 552 (57.0%) | 305 (59.6%) | 138 (54.3%) | 80 (67.8%) | 29 (34.5%) |
| Neither well nor poorly controlled | 213 (22.0%) | 110 (21.5%) | 53 (20.9%) | 26 (22.0%) | 24 (28.6%) |
| Poorly controlled | 50 (5.2%) | 21 (4.1%) | 10 (3.9%) | 9 (7.6%) | 10 (11.9%) |
| Very poorly controlled | 10 (1.0%) | 6 (1.2%) | 3 (1.2%) | 0 (0.0%) | 1 (1.2%) |
