# Supplementary material for: Real‐World Multinational Survey of Chronic Inflammatory Demyelinating Polyneuropathy: Disease Characteristics and Therapeutic Landscape
Source: J Peripher Nerv Syst. 2025 Aug 18;30(3):e70047. doi: 10.1111/jns.70047 (PMC12361836; doi:10.1111/jns.70047)
Supplement: Supplementary file 4 — Supplementary Table 4: Healthcare resource utilization split by region/country. [file JNS-30-0-s002.docx]

## **Supplementary Table 4**: Healthcare resource utilization split by region/country

|  | **All patients** | **Europe** | **US** | **China** | **Japan** |
| --- | --- | --- | --- | --- | --- |
| **HCPs involved in patient management (Top 5), n (%)** | n = 1056 | n = 542 | n = 291 | n = 120 | n = 103 |
| General Neurologist | 882 (83.5%) | 457 (84.3%) | 224 (77.0%) | 120 (100.0%) | 81 (78.6%) |
| Physical Therapist | 399 (37.8%) | 294 (54.2%) | 79 (27.1%) | 15 (12.5%) | 11 (10.7%) |
| Family Doctor/GP/PCP | 364 (34.5%) | 278 (51.3%) | 77 (26.5%) | 6 (5.0%) | 3 (2.9%) |
| Neuromuscular Specialist | 258 (24.4%) | 138 (25.5%) | 77 (26.5%) | 20 (16.7%) | 23 (22.3%) |
| Occupational Therapist | 111 (10.5%) | 67 (12.4%) | 33 (11.3%) | 0 (0.0%) | 11 (10.7%) |
| Internal Medicine/Internist | 87 (8.2%) | 28 (5.2%) | 41 (14.1%) | 15 (12.5%) | 3 (2.9%) |
| Nurse | 87 (8.2%) | 63 (11.6%) | 12 (4.1%) | 2 (1.7%) | 10 (9.7%) |
| Rehabilitation Therapist | 86 (8.1%) | 42 (7.7%) | 15 (5.2%) | 28 (23.3%) | 1 (1.0%) |
| Psychologist | 62 (5.9%) | 49 (9.0%) | 4 (1.4%) | 9 (7.5%) | 0 (0.0%) |
| Psychiatrist | 34 (3.2%) | 25 (4.6%) | 7 (2.4%) | 1 (0.8%) | 1 (1.0%) |
| Ophthalmologist | 16 (1.5%) | 8 (1.5%) | 8 (2.7%) | 0 (0.0%) | 0 (0.0%) |
| Physician associate/assistant | 12 (1.1%) | 12 (2.2%) | 0 (0.0%) | 0 (0.0%) | 0 (0.0%) |
| Speech and Language Therapist | 10 (0.9%) | 6 (1.1%) | 3 (1.0%) | 0 (0.0%) | 1 (1.0%) |
| Neuropsychiatrist | 6 (0.6%) | 4 (0.7%) | 0 (0.0%) | 2 (1.7%) | 0 (0.0%) |
| Other | 13 (1.2%) | 10 (1.8%) | 2 (0.7%) | 0 (0.0%) | 1 (1.0%) |
| **Total number of HCPs involved in management** | n = 1056 | n = 542 | n = 291 | n = 120 | n = 103 |
| Mean (SD) | 2.4 (1.4) | 2.8 (1.5) | 2.0 (1.2) | 2.4 (1.0) | 1.5 (1.1) |
| **Total number of HCP consultations (last 12 months)** | n = 1033 | n = 531 | n = 281 | n = 119 | n = 102 |
| Mean (SD) | 7.7 (12.7) | 9.6 (16.4) | 3.9 (3.7) | 6.8 (4.0) | 9.5 (10.8) |
| **Mobility aids utilized, n (%)** | n = 1056 | n = 542 | n = 291 | n = 120 | n = 103 |
| Cane/walking stick | 373 (35.3%) | 205 (37.8%) | 103 (35.4%) | 36 (30.0%) | 29 (28.2%) |
| Wheeled walker | 85 (8.0%) | 43 (7.9%) | 28 (9.6%) | 10 (8.3%) | 4 (3.9%) |
| Walking frame | 52 (4.9%) | 32 (5.9%) | 8 (2.7%) | 7 (5.8%) | 5 (4.9%) |
| Manual wheelchair | 52 (4.9%) | 20 (3.7%) | 9 (3.1%) | 12 (10.0%) | 11 (10.7%) |
| Motorized wheelchair | 28 (2.7%) | 9 (1.7%) | 9 (3.1%) | 7 (5.8%) | 3 (2.9%) |
| Modified car (e.g. wheelchair accessible vehicle) | 14 (1.3%) | 8 (1.5%) | 4 (1.4%) | 0 (0.0%) | 2 (1.9%) |
| Motorized scooter | 8 (0.8%) | 2 (0.4%) | 5 (1.7%) | 1 (0.8%) | 0 (0.0%) |
| Other | 9 (0.9%) | 8 (1.5%) | 1 (0.3%) | 0 (0.0%) | 0 (0.0%) |
| None | 557 (52.7%) | 285 (52.6%) | 152 (52.2%) | 60 (50.0%) | 60 (58.3%) |
| **Number of hospitalizations (last 12 months), n (%)** | n = 839 | n = 453 | n = 206 | n = 89 | n = 91 |
| 0 | 670 (79.9%) | 395 (87.2%) | 180 (87.4%) | 24 (27.0%) | 71 (78.0%) |
| 1 | 107 (12.8%) | 44 (9.7%) | 21 (10.2%) | 31 (34.8%) | 11 (12.1%) |
| 2 | 35 (4.2%) | 5 (1.1%) | 5 (2.4%) | 17 (19.1%) | 8 (8.8%) |
| 3 | 19 (2.3%) | 6 (1.3%) | 0 (0.0%) | 12 (13.5%) | 1 (1.1%) |
| 4 | 7 (0.8%) | 3 (0.7%) | 0 (0.0%) | 4 (4.5%) | 0 (0.0%) |
| 5 | 1 (0.1%) | 0 (0.0%) | 0 (0.0%) | 1 (1.1%) | 0 (0.0%) |
| **Number of ER admissions (last 12 months), n (%)** | n = 839 | n = 453 | n = 206 | n = 89 | n = 91 |
| 0 | 774 (92.3%) | 422 (93.2%) | 183 (88.8%) | 82 (92.1%) | 87 (95.6%) |
| 1 | 54 (6.4%) | 27 (6.0%) | 19 (9.2%) | 6 (6.7%) | 2 (2.2%) |
| 2 | 9 (1.1%) | 2 (0.4%) | 4 (1.9%) | 1 (1.1%) | 2 (2.2%) |
| 3 | 2 (0.2%) | 2 (0.4%) | 0 (0.0%) | 0 (0.0%) | 0 (0.0%) |
